# ACE2, a drug target for COVID-19 treatment?

**DOI:** 10.1007/s11845-022-03055-1

**Published:** 2022-06-14

**Authors:** Yang Liu, Huilian Huang

**Affiliations:** 1 The Department of Anesthesia, Huzhou Central Hospital, Affiliated Central Hospital Huzhou University, 1558 Sanhuan North Road, Huzhou, 313000 Zhejiang People’s Republic of China; 2 Key Laboratory of Molecular Medicine, Huzhou Central Hospital, Affiliated Central Hospital Huzhou University, 1558 Sanhuan North Road, Huzhou, 313000 Zhejiang People’s Republic of China

To the editor:

The SARS-CoV-2 virus is responsible for causing the current global COVID-19 pandemic, which has lasted for over 2 years now. Since its first outbreak at the end of the year 2019, the SARS-CoV-2 virus has undergone multiple mutations, from the Alpha variant to the Omicron variant, which is currently the most prevalent one in the world. With each mutation, the virus tends to become further infectious, raising health-related concerns. Each mutation leads to a shorter infection time and increased occult. According to the global statistics, the number of people that have been infected with the SARS-CoV-2 virus to date is approximately 500 million, and the number continues to increase sharply to several millions of infections as time passes [1].

Previous studies have confirmed that ACE2 is the “key” factor involved in the process of entry of SARS-CoV-2 into the host cells. In the initial step of viral infection, the virus binds to the ACE2 receptor via the RBD domain in the viral spike protein [2]. The specific binding of the RBD domain to the cell surface receptor ACE2 induces the cleavage of the S protein by the cell surface protease TMPRSS2 [3]. This step separates the S1 fragment from ACE2, exposing the internal fusion peptide of the S2 fragment, which then inserts into the host cell membrane [4].

Lubin et al. reported that the RBD domain in the Omicron variant is highly flexible and unstable [5] and forms an RBD dimer with the adjacent RBD domain, rendering it easier for the S protein to maintain an open conformation. This step significantly enhances the binding force between RBD and the ACE2 receptor by approximately ten times that observed in the original strain of this virus. Woo et al. reported that the mutation of T478K, Q493K, and Q498R led to an increase in the virulence of the Omicron variant [6]. According to most of the current studies, the RBD domain of the Omicron variant exhibits a greater vital binding force toward ACE2, which also increases the infectivity of this variant to a certain extent. These mutations of the Omicron variant led to several significant changes in its antigenicity, enabling better immune escape and protection against most of the current vaccinations against the virus [7].

Perhaps the only thing to be thankful for regarding these mutations is that the clinical manifestations of the Omicron variant are much less severe compared to the Delta variant. According to a study conducted in the USA on the Omicron infection in children under the age of 5 years, the emergence of the Omicron variant decreased the number of emergency visits by 29% compared to the Delta variant, while the number of hospitalizations decreased by 67% and the number of hospitalizations involving admission into the ICU decreased by 68% [8]. These statistics are consistent with the findings of another study, which reported that the symptoms observed in the patients infected with the Omicron variant were milder compared to those observed in the patients with Delta variant infection [9]. This has greatly reduced the heavy burden on the medical system. However, if the total number of infections increases, the final number of hospitalizations would be alarming once again. For instance, the number of deaths due to infection with the Omicron variant during the fifth wave in Hong Kong has exceeded the sum of the deaths during the previous four waves of the virus [10].

The virus has been evading the resistance of the vaccine through continuous mutation, thereby troubling scholars as to how to prevent the virus from invading the human body. The development of specific drugs against the COVID-19 virus continues to be one of the most urgent problems to be resolved. Currently, Paxlovid, a small molecule drug developed by Pfizer, is exhibiting excellent therapeutic effect on COVID-19 patients. Paxlovid reduces the risk of hospitalization or death related to COVID-19 disease by 89% [11]. Paxlovid inhibits the replication of the virus in the human body by targeting the M protein of SARS-CoV-2, although it does not eliminate the virus entirely. Therefore, this drug is suitable for the early stages of COVID-19 infection. The BRII-196/BRII-198 monoclonal antibody developed by China Tengsheng Huachuang Pharmaceutical Technology Company reportedly reduces the risk of hospitalization or death related to COVID-19 disease by 80% [12]. The BRII-196/BRII-198 monoclonal antibody effectively neutralizes the virus and prevents it from invading the human body. However, this antibody is highly specific in viral recognition, and, therefore, if the virus mutates further, the antibody would be rendered ineffective.

In a previous article reported by our research group, the essential role of soluble ACE2 or ACE2-specific small molecular peptides targeting ACE2 in preventing the virus from entering the human body was summarized, thereby suggesting ACE2 as the potential target for the development of specific drugs against the COVID-19 virus [13]. Although it is feared that both Alpha (the earlier variant) and Omicron (the current variant) variants may mutate and become capable of escaping the immunity elicited by antibodies, the virus would continue to have a binding affinity for ACE2, and the binding force between the virus and ACE2 is expected to become stronger with each mutation [14, 15]. ACE2 has been consistently recognized as the first barrier for viruses to invade when they enter the human body. Therefore, maintaining this barrier to prevent viruses from entering the human body should be the objective of the research works aimed at developing novel and specific drugs against the COVID-19 virus. Several previous studies have reported that soluble ACE2 could serve as a candidate drug against the COVID-19 disease [16], although further evidence would better validate such findings. Moreover, the essential role of ACE2 protein in the regulation of cardiovascular functioning renders it further crucial to maintain caution when using this protein as a drug target. The scholars are reluctant to eliminate ACE2 as a drug target, though. This is because ACE2 would remain an essential channel for the COVID-19 virus to enter the human body despite any number of mutations. The hope of closing this channel and achieving effective treatment of the COVID-19 disease, therefore, continues.
